# Factors on patency periods of subcutaneous central venous port: long-term results of 1,408 patients

**DOI:** 10.1186/1470-7330-15-S1-P27

**Published:** 2015-10-02

**Authors:** BK Aribas, T Uylar, MY Aksoy, I Turker, F Yildiz, R Tiken, I Akdulum

**Affiliations:** 1 Dr. Abdurrahman Yurtaslan Ankara Oncology Education and Research Hospital, Ankara, Turkey

## Aim

To examine factors on patency times including complications of subcutaneous venous chest ports insertion using ultrasonography guidance in 1,408 patients with long-term follow-up.

## Patients and methods

Between April 2009 and March 2014, subcutaneous venous chest ports were placed in 1,408 patients, 574 women and 834 men, mean age of 55.4±12 1 years. Factors on patency times and complications rates of ports were compared. Age, gender, access, site of malignancy, and coagulation parameters were variables and multivariable Cox regression test was used. The successes of jugular and subclavian groups were compared by univariate Kaplan-Meier survival analysis, also. Port was used for treatment after 3 hours on the procedure day.

## Results

Fifty-seven patients underwent port removal due to complications. As a rate in 100 catheter days, ports were explanted in 29 (0.0054) due to thrombosis, in 9 infection (0.0017), in 8 (0.0015) for catheter malposition, in 5 bleeding (0.0009), in 5 skin necrosis with infection in one (0.0009), one port reservoir flip-over (0.0002), total 57 patients (0.0107). Patency times were not different in jugular and subclavian veins (p=0.230). Any factor was not significant except for malignancy site (p=0.002).

## Conclusion

There was no significant difference of factors on patency times including complications in jugular vein access or subclavian vein access using ultrasonography. Malignancy site was the only significant factor in success. Malignancy site and gender were significant factors in thrombosis, as significantly higher in extremity and involving > one regions and in female patients.

